# Investigation of the Effect of Isotonic Drinks on Quantitative MRI Markers of the Liver and Spleen

**DOI:** 10.1002/nbm.70156

**Published:** 2025-10-08

**Authors:** Natassa N. Pittas, Michael Pavlides, Ferenc E. Mózes

**Affiliations:** ^1^ Department of Oncology University of Oxford Oxford UK; ^2^ Oxford Centre for Clinical Magnetic Resonance Research, Radcliffe Department of Medicine University of Oxford Oxford UK; ^3^ Translational Gastroenterology Unit University of Oxford Oxford UK; ^4^ Oxford NIHR Biomedical Research Centre Oxford University Hospitals NHS Foundation Trust and the University of Oxford Oxford UK

## Abstract

Quantitative MRI markers of the liver are attractive diagnostic tools of metabolic dysfunction‐associated steatotic liver disease (MASLD). However, $T_{1}$ measurements are confounded by physiological factors, such as hydration. The aim of this study is to evaluate the dynamic effects of hydration with isotonic drinks on quantitative MRI measurements of the liver in healthy male individuals. Shortened modified Look‐Locker inversion recovery‐based (shMOLLI) $T_{1}$, ${T_{2}}^{*}$, proton density fat fraction (PDFF), apparent diffusion coefficient (ADC), stiffness, and volume of the liver, as well as shMOLLI $T_{1}$ and volume of the spleen were measured repeatedly in six healthy male volunteers at 3 T after ingesting 0.5, 1.0, and 1.5 L of isotonic drink on three different days. $T_{1}$ data were fitted to a first‐order linear time‐invariant system model, descriptive statistics were calculated for the other parameters, and linear mixed models were employed to assess co‐variates of quantitative MRI parameters. Liver $T_{1}$ showed a maximum absolute increase of 58, 60, and 90 ms, while spleen $T_{1}$ showed a maximum absolute increase of 45, 70, and 150 ms corresponding to 0.5, 1.0, and 1.5 L of isotonic drink, respectively. The maximum change in ADC, ${T_{2}}^{*}$, liver volume and spleen volume gradually increased, while that in liver stiffness gradually decreased with increasing amounts of isotonic drinks. PDFF values had no dependence on the volume of ingested isotonic drink. First‐order models showed increasing time constants for both the liver and the spleen corresponding to increasing isotonic drink volumes. Liver time constants were only dependent on ingested drink volume. This study explored the effect of different volumes of isotonic drink and quantitative liver MRI markers. We found that the increase in $T_{1}$ likely bears clinical significance, however, the variation observed in the other parameters need further investigation and validation in a larger cohort.

## Graphical abstract

Dynamic models relating liver and spleen T_1_ to three different amounts of isotonic drinks (0.5, 1.0, and 1.5 L) are described, and the dependence of liver T_1_, stiffness, volume, and fat fraction on isotonic drinks is discussed. Only T_1_ prolongation bore clinical significance, and the time constants of dynamic models were dependent only on the volume of ingested isotonic drinks.

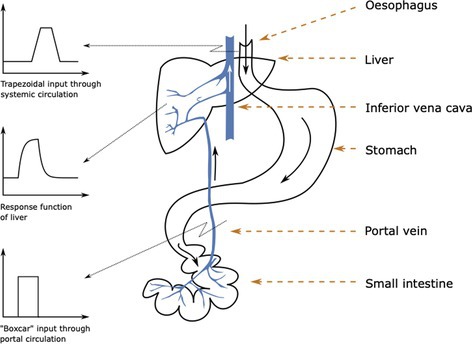

## 1 Introduction

Metabolic dysfunction‐associated steatotic liver disease (MASLD), previously known as non‐alcoholic fatty liver disease (NAFLD) [1], has grown over the past years into a major global health concern, affecting nearly one in three adults worldwide [2]. MASLD encompasses a spectrum of liver conditions and has the potential to progress to cirrhosis and hepatocellular carcinoma (HCC) [3]. In most cases, MASLD correlates with obesity, insulin resistance, and dyslipidemia [4].

Diagnosing and monitoring MASLD remains a challenge. Currently, liver biopsy is still considered the gold standard diagnostic method, but it carries risks of bleeding, other complications, or mortality [5]. Its limitations include high intra‐ and inter‐observer variability, high cost, and low spatial coverage of the liver [6]. The increased prevalence of the disease, the disadvantages of biopsy, and the reluctance of individuals to undergo this procedure make non‐invasive diagnostic approaches preferable [7, 8]. As a result, there still is an unmet clinical need for non‐invasive biomarkers to accurately diagnose MASLD, stratify patients, track disease progression, evaluate treatment response, and prognosticate clinical outcomes.

Quantitative MRI markers have garnered increasing attention for their potential to overcome the shortcomings of liver biopsy [9]. In particular, $T_{1}$ mapping has emerged as a promising quantitative MRI technique in the assessment of MASLD [5]. By reflecting the distribution of water molecules in the liver's cells (i.e., hepatocytes) and its extracellular matrix, $T_{1}$ mapping offers insights into the alterations in tissue composition characteristic of MASLD.

While the effects of iron overload [10], steatosis [11], glycogen concentration variations [5], tissue $T_{2}$ [12], magnetization transfer [12], and sequence parameters [13] on $T_{1}$ mapping have been described previously, other physiological processes, such as hydration effects remain understudied in the liver. A recent study found that acute hydration did not result in clinically significant changes in $T_{1}$ and $T_{2}$ measurements of the human myocardium [14]. Unlike the myocardium, the liver is capable of large dynamic changes as a response to acute physiological triggers, suggesting that hydration might have clinically relevant effects on quantitative MRI measurements.

Thus, the aim of this work was to evaluate the dynamic effects of hydration with isotonic drinks on quantitative MRI measurements of the liver in healthy male individuals. The primary objective was to evaluate the dynamic response of $T_{1}$ measurements to various volumes of isotonic drinks, while the secondary objectives were to evaluate the response of ${T_{2}}^{*}$, liver stiffness measured by magnetic resonance elastography (LSM‐MRE), apparent diffusion coefficient (ADC), proton density fat fraction (PDFF), and liver volume measurements. The secondary objectives also included the characterization of the dynamic response of spleen to isotonic drinks using the same quantitative MRI markers as mentioned before.

## 2 Theory

Linear time invariant (LTI) dynamic systems are widely used in modeling mechanical or electrical processes. Such models can also be applied to biological systems when there are no competing effects (or they are negligible) and in the presence of only marginal diurnal variation.

Among the many functions of the liver, storage is an important one: carbohydrate, iron, and even water storage as a result of increased blood volume are well‐understood processes. Since first‐order dynamic LTI systems are well‐suited for characterizing systems with storage capacity, we considered the liver's ability to change its $T_{1}$ as governed by a first‐order ordinary differential equation. A typical first‐order process can be described by Equation 1, where $u\left(t\right)$ describes the perturbation or input signal to the system and $y\left(t\right)$ is the response of the system characterized by the time constant τ.

(1) $$\tau \frac{\mathrm{dy}\left(t\right)}{\mathrm{dt}}+y\left(t\right)=u\left(t\right)$$

As long as the input function $u\left(t\right)$ is known, $y\left(t\right)$ can be determined from the Laplace‐transformed version of Equation 1, as given by Equation 2.

(2) $$Y\left(s\right)=U\left(s\right)\frac{1}{\mathrm{\tau s}+1}$$

**FIGURE 1:**
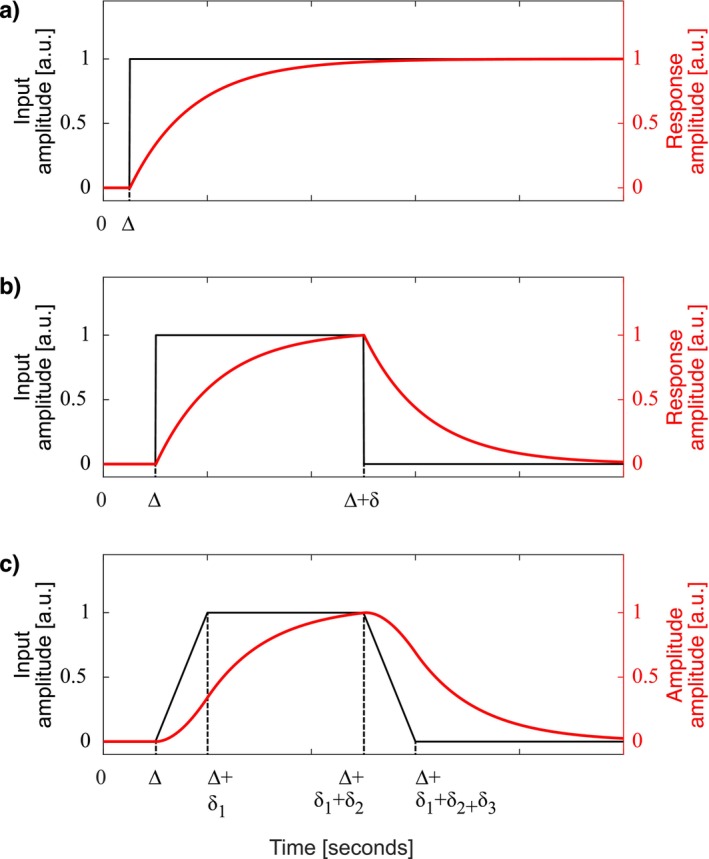
Example input functions (in gray) and the corresponding response (in red) of a first‐order linear time‐invariant dynamic system: (a) delayed step or Heaviside input function with Δ delay time; (b) rectangular or top hat input function with Δ delay and δ duration; (c) trapezoidal input function with a delay time Δ, ramp‐up time $\delta_{1}$, flat top time $\delta_{2}$, and ramp‐down time $\delta_{3}$. Note the damped shape of the response function in all three cases.

In a well‐controlled experiment, such as the one described in the current study, simple input functions can be assumed. Figure 1 shows three simple input function shapes and their corresponding responses given by a first‐order LTI system. Of these, Figure 1a shows the response to possibly the simplest input, the step function, and Figure 1b,c shows the responses to a rectangular and a trapezoidal input and are used in this work. The analytical form of the system's response function is typically determined in the Laplace domain in the presence of a given or assumed input function. The parameters of the system, as well as those of the input function, can be estimated by fitting time series data to the analytical form of the response function.

## 3 Methods

### 3.1 Participant Selection

This study recruited six healthy male participants aged 18–60 years, with a body mass index of 18.5–30 kg/m^2^, and implemented water ingestion protocols to stimulate liver $T_{1}$ response. Exclusion criteria were as follows: female sex, presence of pacemakers, metallic implants, or any other contraindication for MRI, regular consumption of alcohol exceeding 14 units/week, or self‐reported history of heart, lung, liver, or kidney disease or diabetes. Only male participants were included as significant differences were highlighted in liver $T_{1}$ measurements across demographic groups, particularly between pre‐menopausal women, post‐menopausal women, and age‐matched men. Pre‐menopausal women exhibit longer liver $T_{1}$ compared to the other groups [15].

The study was conducted in accordance with the ethical guidelines of the 1975 Declaration of Helsinki and was approved by a subcommittee of the University of Oxford Central University Research Ethics Committee (reference: R92109/RE001). All participants gave written informed consent.

### 3.2 Study Protocol

**FIGURE 2:**
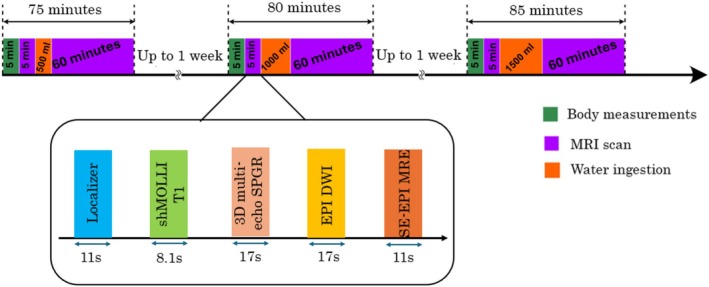
Study flow chart outlining the order and timing of various activities involved in the study, including body measurements, MRI scans, and isotonic drink consumption. The sequences used for the baseline (pre‐ingestion) measurements are shown in the box under the timeline.

Participants were given 500, 1000, and 1500 mL of isotonic drink orally during three study visits taking place on different days. Isotonic drinks were prepared using SIS Hydro (Science in Sport, Milan, Italy) electrolyte tablets dissolved in bottled still mineral water. During each study visit, participants had a baseline MRI scan including parametric maps. This baseline scan was followed by the ingestion of 500, 1000, or 1500 mL of isotonic drink, followed by approximately 50–60 more minutes of MRI scanning. Parametric maps were collected several times over this scan. Figure 2 illustrates the timing of study interventions.

### 3.3 MRI Data Acquisition

All MR scans were performed with the participants lying supine in a 3 T Prisma MRI scanner (Siemens Healthineers, Erlangen, Germany) using non‐invasive techniques. A body array coil with 18 channels and a spine array coil with 32 channels were used. The individual components of the MR protocol were liver $T_{1}$, liver ${T_{2}}^{*}$, liver PDFF, liver volume, liver ADC, liver MRE, spleen $T_{1}$, and spleen volume.

Table 1 shows sequence parameters and timings of each acquisition post‐ingestion. A justification of the choice for the sampling interval is provided in the Supplementary Materials.

**TABLE 1:** Parameters and acquisition frequency for MRI sequences used in the protocol.

Sequence	shMOLLI	mDIXON (6 TEs)	Diffusion (DWI)	MRE
Parameter measured	$T_{1}$	${T_{2}}^{*}$, PDFF	ADC	LSM‐MRE
Acquisition frequency	Every 5 min	Every 15 min	Every 15 min	Every 30 min
Number of slices	1	64	13	4
Slice thickness (mm)	8.0	4.0	6.0	8.0
TR (ms)	2.40	9.00	1600	1000
TE (ms)	1.01	${\mathrm{TE}}_{1}=1.06$	37.0	47.0
		ΔTE = 1.14		
TI (ms)	100, 100 + RR, 100 + 2RR, 100 + 3RR, 100 + 4RR, 180, 260	—	—	—
Flip angle (degrees)	35	4	90, 180	90
Acceleration type and factor	GRAPPA 2	CAIPIRINHA 1 + 2	GRAPPA 2	GRAPPA 2
Bandwidth (Hz/px)	1302	1040	2084	2170
b‐values (s/mm^2^)	—	—	0, 200, 600	—
FOV (mm)	360 × 270	400 × 330	414 × 365.5	420 × 420
Matrix size	144 × 192	106 × 160	106 × 120	128 × 128
Acquisition time (s)	8.1	17	17	11

Abbreviations: ADC: apparent diffusion coefficient; DWI: diffusion‐weighted imaging; FOV: field of view; LSM: liver stiffness measurement; mDIXON: multi‐echo DIXON; MRE: magnetic resonance elastography; PDFF: proton density fat fraction; shMOLLI: shortened modified Look‐Locker inversion recovery; TE: echo time; TI: inversion time; TR: repetition time.

ShMOLLI $T_{1}$ values were adjusted for iron and fat content, as described in [13].

${T_{2}}^{*}$ and PDFF maps were reconstructed using the MAGO algorithm [16]. Volumes were estimated via semi‐automated segmentation in ITK‐SNAP (version 4.2.0, April 22, 2024) [17] by using slice‐by‐slice double‐threshold masks with an active contour algorithm that employed several seeds depending on the size of the liver. Blood vessels were excluded from the final liver volume.

**FIGURE 3:**
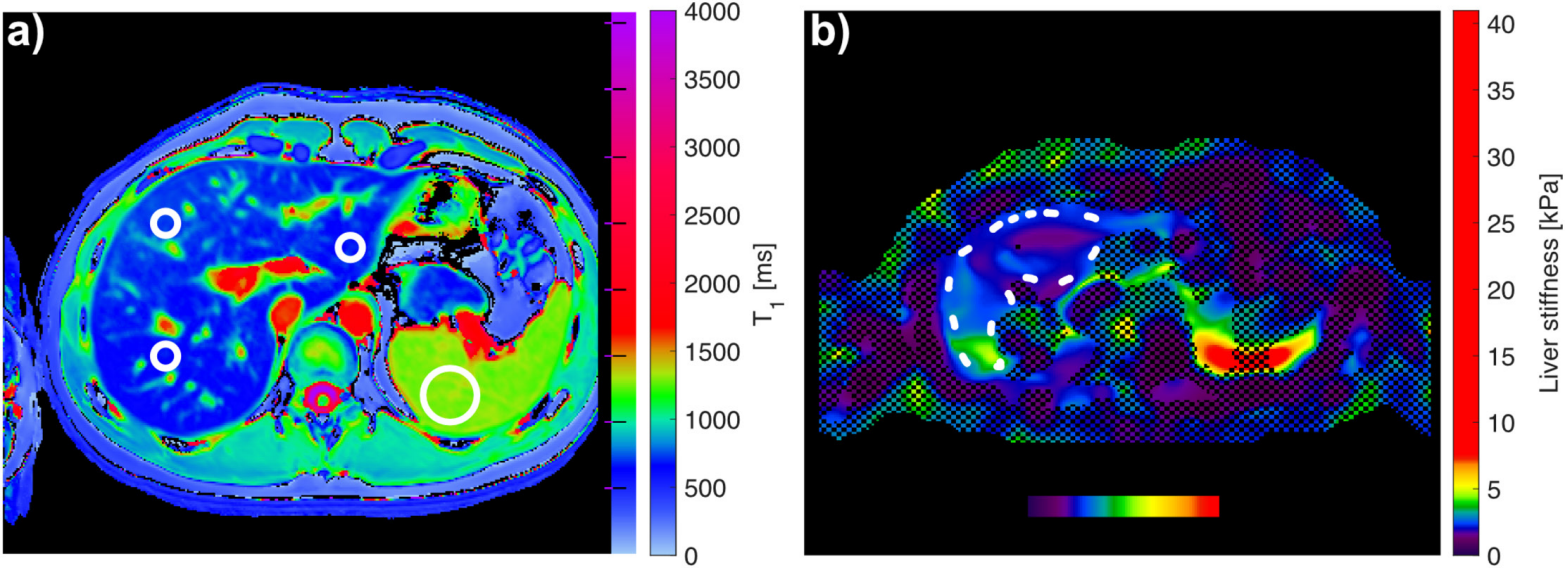
Example placement of circular regions of interest (ROIs) within the liver and spleen for $T_{1}$, PDFF, and ADC maps (a). Large, organ‐wide ROIs were used to determine liver stiffness (b).

Average $T_{1}$ and ADC values were determined using the MicroDICOM DICOM Viewer (version 2024.1, February 26, 2024), while MATLAB analysis was employed to determine MRE liver stiffness, PDFF, and ${T_{2}}^{*}$ values. MRE‐based liver stiffness measurements were determined according to the recommendations of the Quantitative Imaging Biomarker Alliance (QIBA) MRE profile [18]. Region of interest (ROI) analysis was performed on the collected maps at each time point for each isotonic drink volume using in‐house MATLAB (MathWorks, Natick, MA, USA) scripts. ROIs were placed within the liver: two ROIs were positioned in the right lobe—one in the posterior region and one in the anterior region—, while the third ROI was placed in the left lobe when this was visible. A single ROI was placed in the posterior part of the spleen. Figure 3 illustrates the placement of ROIs in the liver and the spleen.

Average values were calculated from all ROIs for the parameters mentioned above.

Subsequently, $T_{1}$ measurements in the liver and spleen were fitted to first‐order dynamic system models, assuming top hat and trapezoidal input functions. New parameters were generated from these models to estimate water uptake rates in the systemic and portal hepatic circulation in response to the ingested amounts of isotonic drink.

### 3.4 Modelling and Statistical Analysis

**FIGURE 4:**
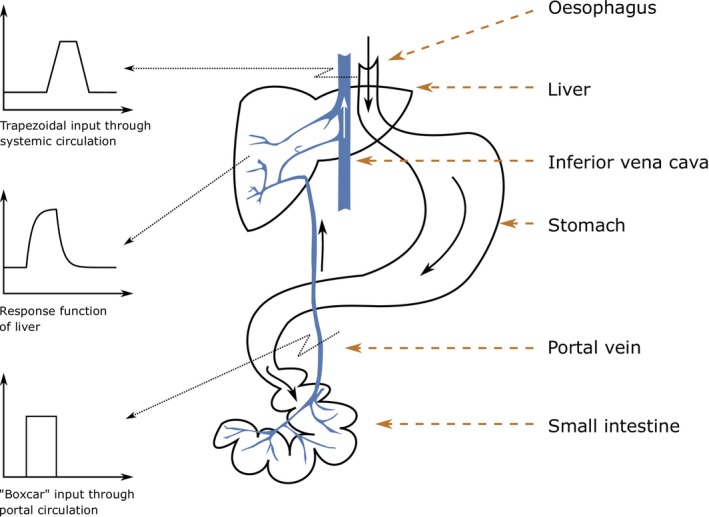
Water distribution in the body after water ingestion.

Previous work suggests that both the liver and other organs' $T_{1}$ respond to acute hydration in a dampened‐fashion, i.e., as first‐order linear time‐invariant systems [19]. Figure 4 shows the distribution and the travel path of liquids within the body after oral ingestion. Briefly, after ingestion, liquids pass through the esophagus and enter the stomach from where they are emptied into the small intestine. Liquids are then absorbed into the bloodstream through the walls of the intestine. Absorbed liquids enter the bloodstream and are carried by the portal vein to the liver. After passing through the liver, the blood, which now contains the absorbed liquids, is transported to the hepatic veins, which drain into the inferior vena cava and reach the heart. Afterwards, oxygenated blood, mixed with the absorbed liquid, is distributed via the systemic circulation to various organs and tissues throughout the body, including the spleen.

To determine the response functions for the liver and spleen $T_{1}$ values, a method based on the transformation of first‐order differential equations using the Laplace transform was employed. Initially, the first‐order differential equations describing the system were transformed into the Laplace domain. The Laplace‐transformed equations were then used to derive the response of the system to different input functions and then inverse Laplace transformed the response into the time domain. The use of rectangular function as an input to the liver is warranted as the increase in fluid volume reaching the liver from the small intestine can be considered as a prolonged bolus. This approach extends the concept of arterial input function known from dynamic contrast enhanced MRI measurements employing short contrast agent boluses [20]. Since the spleen receives the increased fluid volume in the blood over a prolonged time and not as suddenly as the liver does from the small intestine, the rectangular function needs a small adjustment to account for the delayed onset of maximal blood volume change, leading to the use of the delayed trapezoidal input function.

Water that has been consumed, passed through the stomach, partially released in the portal circulation, and absorbed in the small intestines was assumed to behave as a rectangular input function. In this case, liver $T_{1}$ is described by Equation 3:

(3) $$T_{1}\left(t\right)=k_{l}\left(1-e^{-\frac{t-\Delta }{\tau_{l}}}\right)u\left(t-\Delta \right)-k_{l}\left(1-e^{-\frac{t-\Delta -\delta }{\tau_{l}}}\right)u\left(t-\Delta -\delta \right)+T_{1}\left(0\right)$$

where u(t) is the Heaviside step function [21], $k_{l}$ is a gain factor which is related to the magnitude of the change of $T_{1}$, $\tau_{l}$ is the time constant of the liver which determines the rate of water uptake, Δ is the delay between ingesting the isotonic drink and the initial upslope in $T_{1}$, δ is the length of the pulse input, and $T_{1}$(0) is the baseline $T_{1}$ measurement in the absence of the isotonic drink [19].

Spleen $T_{1}$ measurements were fitted to Equation 4, which corresponds to the response to a trapezoidal input function:
(4)

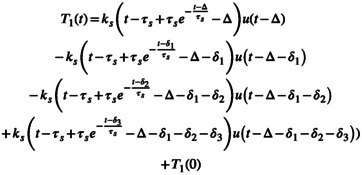

where Δ is the delay between the start of isotonic drink ingestion, $\delta_{1}$ is the duration of the trapezoidal up‐slope, $\delta_{2}$ is the duration of the trapezoidal flat‐top, and $\delta_{3}$ is the duration of the trapezoidal down‐slope, as shown on Figure 1c.

Paired sample *t*‐tests were employed to compare measurements taken at baseline (i.e., before the isotonic drink ingestion) with those taken at the point where measured parameters reached their maximum. Paired sample t‐tests were conducted for liver $T_{1}$, ADC, LSM, PDFF, and spleen $T_{1}$. For all statistical methods, *p*‐values less than 0.05 were considered as evidence of statistical significance.

The parameters gained from the time series fitting (Equation 3 and Equation 4) were used as response variables in linear mixed models (LMMs). The fixed effects were the volume of isotonic drink (0.5, 1.0, and 1.5 L), age, BMI, baseline liver or spleen volume, and baseline IVC/Ao ratio of each participant; and participants were considered as random effects. The LMM was fitted using the *‘mixedlm’* function from the ‘*statsmodels*’ library in Python.

## 4 Results

Table 2 presents a summary of the mean maximum change in various parameters for the three isotonic drink volumes.

**TABLE 2:** Mean maximum absolute and relative change in quantitative MRI parameters and *p*‐values arising from paired *t*‐tests.

Quantitative marker	0.5 L			1.0 L			1.5 L		
	Absolute ↑	Relative ↑	*p*‐Value	Absolute ↑	Relative ↑	*p*‐Value	Absolute ↑	Relative ↑	*p*‐Value
Liver									
$T_{1}$ (ms)	58 ± 24	8% ± 3%	0.0028	60 ± 26	8% ± 3%	0.0033	90 ± 9	12% ± 1%	0.00004
${T_{2}}^{*}$ (ms)	2.5 ± 0.6	9% ± 3%	0.0225	3.2 ± 0.7	12% ± 2%	0.0026	3.3 ± 0.2	12% ± 1%	0.0001
Volume (mL)	90 ± 40	5% ± 2%	0.0701	120 ± 20	8% ± 2%	0.0042	140 ± 60	8% ± 2%	0.0548
ADC (mm^2^/s)	90 ± 10	9% ± 1%	0.0008	140 ± 30	16% ± 4%	0.0052	200 ± 60	20% ± 7%	0.0324
PDFF (%)	0.07 ± 0.03	7% ± 5%	0.4530	0.09 ± 0.05	9% ± 7%	0.2434	0.07 ± 0.03	5% ± 2%	0.2187
LSM (kPa)	−0.4 ± 0.1	−13% ± 3%	0.0091	−0.45 ± 0.09	−16% ± 3%	0.0002	−0.5 ± 0.1	−17% ± 4%	0.0089
Spleen									
$T_{1}$ (ms)	45 ± 7	3.5% ± 0.5%	0.0009	70 ± 20	5% ± 2%	0.0139	150 ± 50	11% ± 4%	0.045
Volume (mL)	16 ± 5	8% ± 2%	0.0243	21 ± 6	10% ± 3%	0.0241	30 ± 10	17% ± 9%	0.067

**FIGURE 5:**
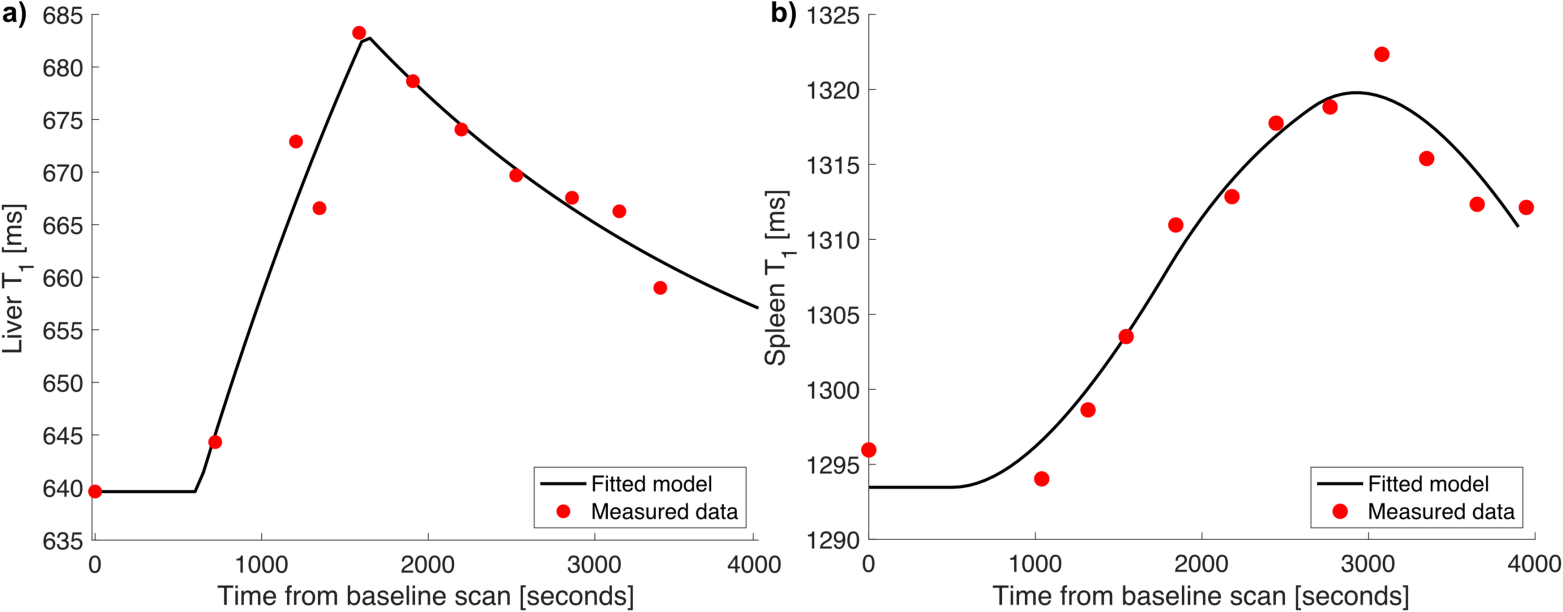
Changes in $T_{1}$ relaxation times of the liver (a), and spleen (b) following isotonic drink ingestion. The red data points represent measured $T_{1}$ values, while the black lines show the fitted curves based on first‐order linear time‐invariant models for each tissue type (Equation 3 for liver and Equation 4 for spleen).

Liver shMOLLI $T_{1}$ values showed a distinct pattern following the ingestion of isotonic drink, as shown on Figure 5a. Specifically, ingestion of 500, 1000, and 1500 mL of isotonic drink led to an increase in liver shMOLLI $T_{1}$, peaking at an average of 24 ± 2, 29 ± 5 and 30 ± 6 min post‐ingestion, respectively. The corresponding absolute increase in liver $T_{1}$ at these time points were 60, 60, and 90 ms. After reaching the peak, there was a gradual decrease in $T_{1}$ time. A similar response was observed in the spleen, where shMOLLI $T_{1}$ values increased similarly, indicating a systemic effect of hydration on $T_{1}$ values, as an additional delay was noted before the up‐slope in $T_{1}$ appeared, as shown in Figure 5b.

The mean maximum increase in liver ${T_{2}}^{*}$ was 2.5 ± 0.6 ms for 500 mL, 3.2 ± 0.7 ms for 1000 mL, and 3.3 ± 0.2 ms for 1500 mL of isotonic drink. Liver ADC showed an average maximum increase of 90 ± 10 mm^2^/s for 500 mL, 140 ± 30 mm^2^/s for 1000 mL, and 200 ± 60 mm^2^/s for 1500 mL. PDFF values showed no statistically significant effect of isotonic drink intake on liver fat measurements.

Isotonic drink ingestion also had an impact on liver and spleen volumes. An increase in the volumes of both the liver and spleen corresponded to increasing volumes of consumed isotonic drinks. Specifically, after ingesting 500 mL of isotonic drink, liver volume increased by 90 ± 40 mL, while spleen volume increased by 16 ± 5 mL. With 1000 mL of isotonic drink, liver volume showed a rise of 120 ± 20 mL, and spleen volume increased by 21 ± 6 mL. The largest ingested isotonic drink volume was associated with a liver volume increase of 140 ± 60 mL, while spleen volume increased by 30 ± 10 mL. Volume changes in both liver and spleen were significant when compared to baseline.

MRE‐LSM decreased after isotonic drink ingestion. The average absolute value of the difference between the MRE‐LSM measured at the baseline and the MRE‐LSM measured at approximately 30 min after isotonic drink ingestion was 0.4 ± 0.1 kPa for 500 mL, 0.45 ± 0.09 kPa for 1000 mL, and 0.5 ± 0.1 kPa for 1500 mL of isotonic drink, respectively.

**FIGURE 6:**
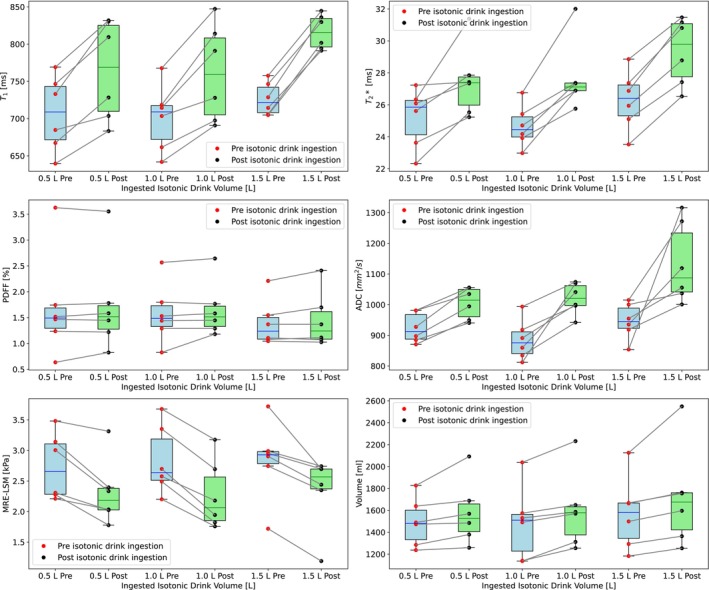
Overview of liver markers showing pre‐ and post‐isotonic drink ingestion data for each participant across different volumes of isotonic drink.

Figure 6 presents an overview of the six markers under investigation for liver: $T_{1}$, ${T_{2}}^{*}$, PDFF, ADC, MRE‐LSM, and volume. The paired box plots represent three pre‐ and post‐intervention data sets for each participant, with the post‐intervention point indicating the maximum change observed for 500, 1000, and 1500 mL ingested isotonic drink, respectively. A similar figure for the spleen is available in the Supplementary Materials.

Table 3 presents the average values of the parameters obtained from fitting the models described by Equation 3 and Equation 4 for each volume of isotonic drink. Model evaluation using LMM was conducted to identify potential co‐variates affecting the model parameters: the volume of isotonic drink, age, BMI, and baseline measurements of IVC/Ao ratio, liver, and spleen volumes. Only the volume of ingested isotonic drink was a significant predictor of the time constant in the liver model (*p* = 0.04). IVC/Ao ratio was a significant predictor of the time constant in the spleen model (*p* = 0.03). Full results of the LMM analysis for all model parameters are provided in the Supplementary Materials.

**TABLE 3:** Results from model fitting of time series Equation 3 and Equation 4.

		$\tau_{l}$ (s)	Δ (s)	δ (s)		
Liver	0.5 L	2614	758	1303		
	1.0 L	3007	821	1534		
	1.5 L	6573	632	1521		
		$\tau_{s}$ (s)	Δ (s)	$\delta_{1}$ (s)	$\delta_{2}$ (s)	$\delta_{3}$ (s)
Spleen	0.5 L	616	609	267	992	129
	1.0 L	1700	745	286	894	232
	1.5 L	2917	568	160	1008	141

## 5 Discussion

Our study explored the relationship between three different volumes of isotonic drink and various quantitative MRI markers measured in the liver ($T_{1}$, ${T_{2}}^{*}$, PDFF, ADC, volume, stiffness) and in the spleen ($T_{1}$, volume). After isotonic drink ingestion there was a delay in $T_{1}$ increase, which was due to the path the isotonic drink followed to reach the liver. Water was absorbed through the walls of the small intestine into the superior mesenteric vein, then into the portal vein, which carried it to the liver. This absorption process took some time, leading to a delay before any changes in $T_{1}$. The isotonic drink was extravasated into the extracellular environment of the liver, leading to an increase in $T_{1}$ values. This increase continued until the liver reached a new equilibrium with the ingested fluid. While not directly comparable with corrected $T_{1}$ (c$T_{1}$), the changes from baseline to peak $T_{1}$ values for all three volumes of isotonic drink were above the 46 ms repeatability coefficient established for c $T_{1}$, potentially reflecting a clinically meaningful change [22]. Liver $T_{1}$ s, then started dropping, eventually returning to baseline levels as the excess water was emptied through the hepatic veins into the inferior vena cava and from there into the systemic circulation, reaching other organs. Our findings are in line with and expand on the previously established relationship between water consumption and liver $T_{1}$ [19, 23].

MRE‐LSM decreased after the isotonic drink ingestion followed by a return to baseline values. While this change was statistically significant, a relative change of 13%, 16%, and 17% corresponding to 500, 1000, and 1500 mL, respectively, was lower than the 19% change defined by the QIBA profile for MRE‐LSM as a minimum amount of change in liver stiffness for it to be considered a true change of clinical utility [18].

ADC values showed an increase with increasing amounts of isotonic drink. This is hardly surprising, as the increased water volume in the extracellular interstitial space contributed to an increased overall diffusivity. The current QIBA profile for DWI only provides a lower limit for detecting real change in ADC for lesions, not for diffuse liver disease; therefore, our findings will need to be revisited once more reproducibility data are available in this space [24].

The study results indicate no statistically significant effect of water intake on PDFF measurements. Across all water volumes, PDFF values remained unchanged for each participant. This consistency was supported by paired t‐tests, demonstrating no significant differences between the values observed at different hydration levels. While this may look reassuring, possibly indicating that water content does not affect PDFF measurements, all volunteers had PDFF ≤ 5% and were not representative of patient populations with MASLD. Further studies involving patients with MASLD are needed to ascertain whether hydration status needs to be a controlled variable when assessing liver fat using PDFF.

The results of the linear mixed model parameters indicated a significant effect of hydration on the liver, particularly on the τ time constant parameter. Hydration is a key driver of changes in liver τ, highlighting the sensitivity of liver $T_{1}$ to alterations in ingested isotonic drink volume. In contrast, hydration did not appear to drive changes in the spleen, perhaps due to the reduction of the water volume in the systemic circulation compared to the splenic circulation by the time it reaches the spleen. Such a reduction in water volume likely reduces the shock to the spleen compared to the liver, which is one of the first organs to encounter the increased blood volume.

Besides the strengths of reporting the relationship between isotonic drink volumes and various quantitative MRI parameters of the liver and spleen, our paper has a number of limitations. Liver $T_{1}$ values did not return to baseline within the one‐hour study period in all participants, possibly due to individual variations in gastric emptying and absorption rates from the gut as each participant responded uniquely to the isotonic drink challenge.

The rate at which water leaves the stomach and is absorbed through the small intestine can vary between individuals due to factors such as intestinal health and baseline hydration status. In some participants, the stomach remained relatively full even after 1 h of MRI scanning, while in others, it emptied rapidly. This difference in gastric emptying times likely impacted how quickly water was absorbed into the bloodstream and reached the liver. Individual differences in baseline hydration status may have also influenced how quickly the liver processed the ingested isotonic drink. Variations in liver size and function can also affect how quickly the liver responds to the ingested isotonic drink, albeit we did not find evidence of this in our linear mixed model. While we uniformly asked participants to refrain from food for 8 h and from water for 30 min before their scans, physiological differences could not be mitigated entirely.

Of note is also the lack of a reproducibility study that assesses the precision of our fitting results. Such a work is required before the wider adoption of our modeling technique.

Furthermore, more precise models could be described by using imaging to measure the portal venous and arterial input functions to the liver and spleen, respectively.

Another important observation in our data is the relatively high baseline MRE‐LSM values recorded before the ingestion of the isotonic drink for some participants. The baseline MRE‐LSM values observed across participants ranged from 1.7 to 3.7 kPa, which exceeds the typical range for healthy individuals—a cut‐off value determined for diagnosing mild fibrosis is 2.65 kPa [25]. Perhaps some of our participants did not have healthy livers, or perhaps the high liver stiffness was caused by the fasting and dehydration before the scan. If so, the decrease in MRE‐LSM after consuming the isotonic drink might actually reflect the liver returning to a normal state rather than just a temporary effect of the drink.

In conclusion, in a response to isotonic drinks consumption only $T_{1}$ showed changes beyond the expected variability, indicating a real, clinically relevant increase. MRE‐LSM remained within the normal variability threshold defined by the QIBA profile, PDFF showed no change, and ${T_{2}}^{*}$ had minimal variation. The significance of ADC and volume changes remain to be investigated in future studies.

While hydration has been shown to significantly impact various quantitative MRI markers, it is important to explore other external factors that may also affect these markers, such as exercise, dietary intake, medications, and diurnal variation in hormone levels. The goal of future studies should be to examine these influences one by one to determine their impact on quantitative liver MRI readings.

## Author Contributions

F.E.M. and M.P. designed the study. N.N.P. and F.E.M. performed data acquisition. N.N.P. performed data analysis and wrote the first draft of this manuscript. All authors approved the final version.

## Conflicts of Interest

N.N.P. and F.E.M. declare no conflicts of interest in relation to the work presented in this manuscript. M.P. is a shareholder of Perspectum Ltd.

## Supporting information


**Figure S1:** Overview of spleen markers showing pre‐ and post‐isotonic drink ingestion data for each participant across different volumes of isotonic drink.
**Table S1:** Results from LMM liver model evaluation.
**Table S2:** Results from LMM spleen model evaluation.

## Data Availability

The data that support the findings of this study are available from the corresponding author upon reasonable request.
