# Supplementary material for: Investigation of the Effect of Isotonic Drinks on Quantitative MRI Markers of the Liver and Spleen
Source: NMR Biomed. 2025 Oct 8;38(11):e70156. doi: 10.1002/nbm.70156 (PMC12508728; doi:10.1002/nbm.70156)
Supplement: Supplementary file 1 — Figure S1: Overview of spleen markers showing pre‐ and post‐isotonic drink ingestion data for each participant across different volumes of isotonic drink. Table S1: Results from LMM liver model evaluation. Table S2: Results from LMM spleen model evaluation. [file NBM-38-e70156-s001.pdf]

Supplementary materials for Investigation of the effect of isotonic drinks on quantitative MRI markers of the liver and spleen

Natassa N. Pittas[1], Michael Pavlides[2,3,4], and Ferenc E. Mózes[2]

[1]Department of Oncology, University of Oxford, Oxford, UK
[2]Oxford Centre for Clinical Magnetic Resonance Research, Radcliffe Department of Medicine, University of Oxford, Oxford, UK
[3]Translational Gastroenterology Unit, University of Oxford, Oxford, UK
[4]Oxford NIHR Biomedical Research Centre, Oxford University Hospitals NHS Foundation Trust and the University of Oxford, Oxford, UK

Justification of T_1 sampling interval

The acquisition frequency of T_1 values can influence fitting results. Given that all acquisitions were performed in breath-hold, our choice of sampling of T_1 values reflect a compromise between participant comfort and a sufficiently high rate of sampling. Additionally, we note that the complete scan protocol also contained measurements unrelated to the liver, which will be reported elsewhere. These additional measurements further constrained our choice of sampling intervals. However, to demonstrate the that a 5-minute T_1 acquisition interval was sufficient, we ran two Monte-Carlo simulations with sampling intervals of 1 minute and 5 minutes, each for two models:

- For a simple exponential model with a time constant: $y(t) = 1 - e^{-t/\tau}$
- For a simplified liver model: $y(t) = (1 - e^{-\frac{t-\Delta}{\tau}})u(t - \Delta) - (1 - e^{-\frac{t-\Delta-\delta}{\tau}})u(t - \Delta - \delta)$, where, for simplicity, we assumed $\Delta = 5$ minutes and $\delta = 20$ minutes, resulting in: $y(t) = (1 - e^{-\frac{t-5}{\tau}})u(t - 5) - (1 - e^{-\frac{t-25}{\tau}})u(t - 25)$

We ran the fitting for τ time constant 200 times in the presence of normally distributed noise and performed a two-sample t-test between the fitting results corresponding to the two sampling schemes for each of the two models. We repeated these simulations for a range of time constants between 1 minute and 10 minutes in steps of 1 minute. Figure 1 shows the p-values from the t-tests and indicate that other than perhaps a 1-minute time constant, the sampling frequency made no statistically significant difference in the fitting result. Since it is hardly conceivable that the time constant of the liver is around 1 minute, we are confident that our sampling did not introduce bias in the fitting results.

Spleen marker changes after isotonic drink ingestion

Fig. 2 presents an overview of the two markers under investigation for spleen: T_1, and volume. The columns represent three pairs of pre- and post-treatment data points for each participant, with the post-treatment point indicating the maximum change observed.

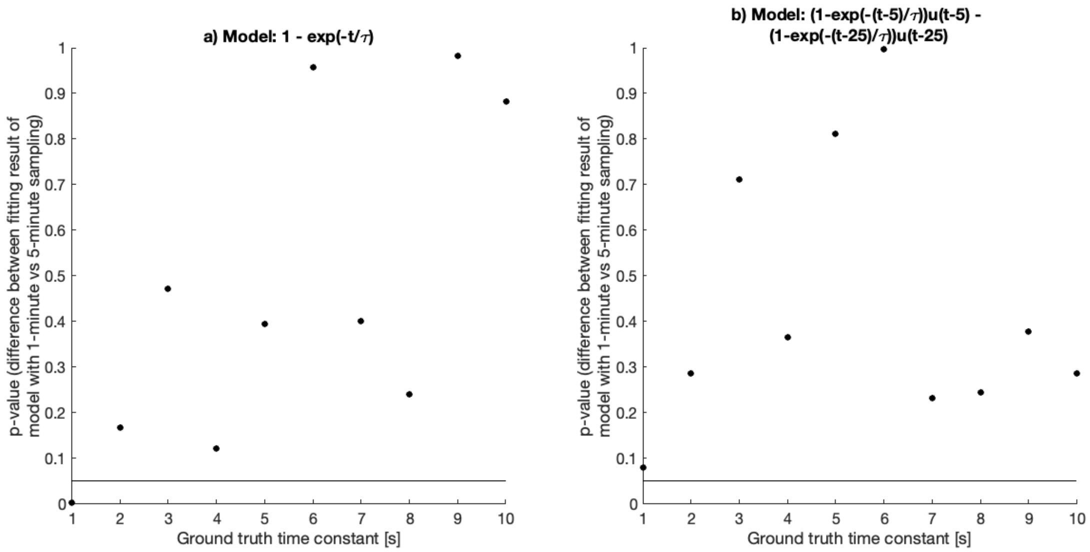

Figure 1: P-values arising from t-tests comparing fitting results from a faster and a slower sampling scheme for different time constants for (a) a simple exponential model and (b) a simplified model of liver T_1.

Linear mixed modeling (LMM) results

LMM was conducted to identify covariates affecting the model parameters: the volume of isotonic drink, age, BMI, and baseline measurements (IVC/Ao ratio and liver/spleen volume). Table 1 and table 2 summarize the results, presenting the coefficients, standard errors, z-scores, and 95% confidence intervals.

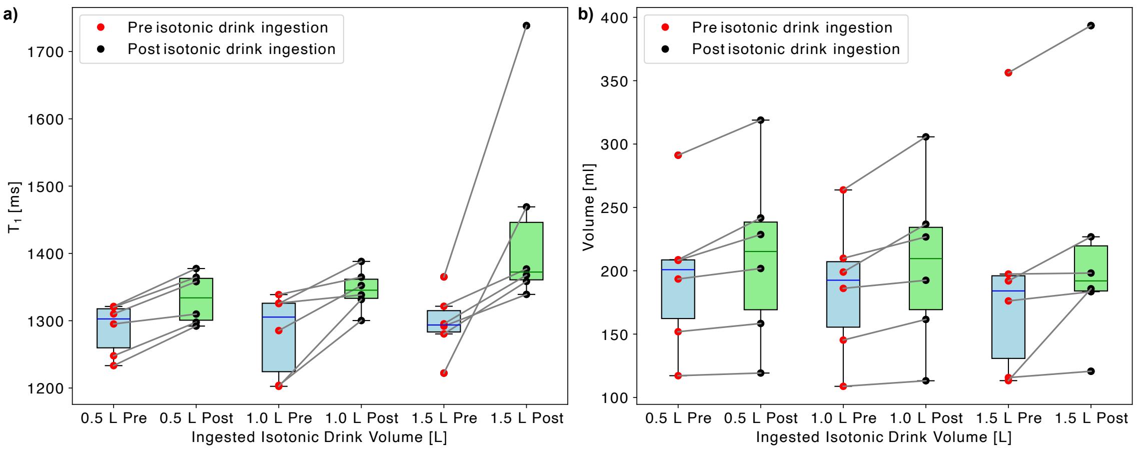

Figure 2: Overview of spleen markers showing pre- and post-isotonic drink ingestion data for each participant across different volumes of isotonic drink: T_1 (a) and spleen volume (b).

Liver time constant - rate of water uptake (τ_l)

	Coef.	Std. Err.	z	P> $\|z\|$	[0.025	0.975]
Intercept	-9860.032	12968.985	-0.760	0.447	-35278.776	15558.712
age	6.071	201.475	0.030	0.976	-388.812	400.954
BMI	-126.175	732.931	-0.172	0.863	-1562.692	1310.343
hydration volume	3611.532	1758.378	2.054	0.040	165.174	7057.889
IVC/Ao	5075.713	4723.334	1.075	0.283	-4181.852	14333.277
Liver volume	0.006	0.005	1.365	0.172	-0.003	0.016
random effect	1682525.609	1747.638				

Liver length of pulse input (δ)

	Coef.	Std. Err.	z	P> $\|z\|$	[0.025	0.975]
Intercept	3597.314	3313.163	1.086	0.278	-2896.366	10090.995
age	59.647	49.849	1.197	0.231	-38.056	157.350
BMI	-131.582	182.179	-0.722	0.470	-488.646	225.483
hydration volume	311.211	360.439	0.863	0.388	-395.236	1017.658
IVC/Ao	141.501	969.531	0.146	0.884	-1758.745	2041.748
Liver volume	-0.001	0.001	-0.973	0.331	-0.003	0.001
random effect	193214.860	552.546				

Liver delay between ingesting the isotonic drink and the initial upslope in $T_1(\Delta)$

	Coef.	Std. Err.	z	P> $\|z\|$	[0.025	0.975]
Intercept	1030.701	1082.734	0.952	0.341	-1091.418	3152.820
age	12.173	16.723	0.728	0.467	-20.603	44.949
BMI	20.303	63.387	0.320	0.749	-103.933	144.538
hydration volume	-95.425	175.713	-0.543	0.587	-439.815	248.966
IVC/Ao	-435.428	462.054	-0.942	0.346	-1341.036	470.181
Liver volume	-0.001	0	-1.486	0.137	-0.001	0
random effect	2427.235	163.316				

Table 1: Results from LMM liver model evaluation

Spleen time constant - rate of water uptake (τ_s)

	Coef.	Std. Err.	z	P> $\|z\|$	[0.025	0.975]
Intercept	-2542.665	2183.523	-1.164	0.244	-6822.292	1736.962
age	7.201	30.964	0.233	0.816	-53.487	67.890
BMI	230.032	141.935	1.621	0.105	-48.156	508.220
hydration volume	-727.602	390.447	-1.864	0.062	-1492.864	37.660
IVC/Ao	-2254.790	1051.556	-2.144	0.032	-4315.802	-193.778
Spleen volume	0	0.003	0.011	0.991	-0.005	0.005
random effect	707.576					

Spleen delay time (Δ)

	Coef.	Std. Err.	z	P> $\|z\|$	[0.025	0.975]
Intercept	465.416	613.422	0.759	0.448	-736.870	1667.701
age	-10.499	8.668	-1.211	0.226	-27.488	6.489
BMI	34.323	39.708	0.864	0.387	-43.504	112.150
hydration volume	-189.173	106.556	-1.775	0.076	-398.018	19.673
IVC/Ao	-433.473	291.298	-1.488	0.137	-1004.407	137.461
Spleen volume	0	0.001	-0.080	0.937	-0.002	0.001
random effect	764.614	64.09				

Spleen ramp-up time (δ_1)

	Coef.	Std. Err.	z	P> $\|z\|$	[0.025	0.975]
Intercept	1840.185	2309.280	0.797	0.426	-2685.922	6366.291
age	-5.979	30.573	-0.196	0.845	-65.901	53.943
BMI	16.357	138.313	0.118	0.906	-254.731	287.445
hydration volume	385.097	267.156	1.441	0.149	-138.519	908.714
IVC/Ao	-258.273	725.823	-0.356	0.722	-1680.860	1164.314
Spleen volume	-0.002	0.003	-0.748	0.454	-0.008	0.004
random effect	107858.814	440.759				

Spleen flat top time (δ_2)

	Coef.	Std. Err.	z	P> $\|z\|$	[0.025	0.975]
Intercept	1880.369	2404.923	0.782	0.434	-2833.192	6593.931
age	-41.843	32.980	-1.269	0.205	-106.483	22.796
BMI	142.360	150.261	0.947	0.343	-152.146	436.866
hydration volume	19.789	372.603	0.053	0.958	-710.501	750.078
IVC/Ao	-1429.416	973.838	-1.468	0.142	-3338.104	479.272
Spleen volume	-0.002	0.003	-0.606	0.545	-0.007	0.004
random effect	51269.024					

Spleen ramp-down time (δ_3)

	Coef.	Std. Err.	z	P> $\|z\|$	[0.025	0.975]
Intercept	-3264.909	4013.259	-0.814	0.416	-11130.753	4600.934
age	-51.904	56.267	-0.922	0.356	-162.186	58.378
BMI	171.016	257.520	0.664	0.507	-333.713	675.746
hydration volume	660.227	683.855	0.965	0.334	-680.104	2000.559
IVC/Ao	554.389	1875.956	0.296	0.768	-3122.417	4231.196
Spleen volume	0.009	0.005	1.675	0.094	-0.002	0.020
random effect	50085.281	891.410				

Table 2: Results from LMM spleen model evaluation
